# Survey and analysis of the availability and affordability of essential drugs in Hefei based on WHO / HAI standard survey methods

**DOI:** 10.1186/s12889-020-09477-9

**Published:** 2020-09-15

**Authors:** Zuojun Dong, Qiucheng Tao, Guojun Sun

**Affiliations:** Zhejiang University of Technology, Hangzhou, Zhejiang China

**Keywords:** Hefei, Essential drugs, Availability, Affordability

## Abstract

**Background:**

The 2018 edition of the National Essential drugs List came into effect in September 2018. Relevant policies require that all primary medical and health institutions should supply national essential drugs, while secondary and tertiary medical and health institutions should supply a certain percentage of essential drugs.

**Methods:**

Our research used the standard methods of WHO and HAI, selected 50 basic drugs, combined with the actual situation of Hefei City, selected 30 medical institutions and 30 pharmacies, conducted drug availability and affordability studies.

**Results:**

The availability of the lowest-price generics (LPGs) of essential drug in Hefei is much higher than that of the Originator brands (OBs); the overall affordability is better, but there is a large gap between the affordability levels of OBs and LPGs. OBs are relatively poorly affordable.

**Conclusion:**

The implementation of the national essential drug system in Hefei has achieved certain results, but there is still a gap from the expected target. It is recommended to update and adjust the list of essential drugs in accordance with the demand for clinical medicines, ensure that medical and health institutions at all levels supply essential drugs, popularize knowledge of essential drugs, and moderately adjust the price of OBs to reduce the medication burden.

## Background

WHO proposes that essential medicines should be necessary for public health, and should be accessible at all times, and be applied to all levels of society in appropriate dosage forms. However, according to the World Health Report 2000, about one-third of the world’s population still lacks access to essential medicines. Despite some health policies aimed at addressing high levels of medical expenses, the high cost of medical services and medicines is still considered to be the main obstacle to access to health care. In 2003, the World Health Organization and the International Health Action (HAI) published a manual describing standard methods to measure drug prices to help policy makers clearly identify purchase prices and identify any major issues in their policies. Therefore, medical institutions at all levels should ensure the accessibility of essential medicines, especially essential medicines for the treatment of common diseases and high incidence [1].

Due to the rapid economic development and increasing government investment in health in recent decades, the Chinese government is working hard to revitalize the health care network. However, the improvement of sanitation conditions (such as infrastructure and human resource development) has not directly translated into improved sanitation. The burden of disease, especially among rural residents, is still very high; people continue to complain that medical expenses are skyrocketing, and medical insurance is not included, so they must pay for it at their own expense. The China Health Survey shows that 30.3% of rural residents in 2003 and 24.7% of rural residents disregarded doctors’ hospitalization recommendations, mainly in terms of economic affordability [2]. In 2018, the Chinese government adjusted the list of essential medicines. After the adjustment, the number of varieties has increased. This adjustment should better meet the daily treatment needs of patients. The Chinese government attaches great importance to the shortage of drugs and has issued several documents to guide the government’s centralized drug procurement. In January 2019, the State Council of China issued the “Pilot Program for Centralized Procurement and Use of Drugs in China”, marking the official start of China’s centralized drug procurement. The implementation of the basic medicine system has played an important role in promoting the comprehensive reform of grass-roots medical and health institutions, and stipulates that all basic medicine and health institutions are equipped with basic medicines. Therefore, basic medicines directly affect the availability and affordability of medicines.

At present, many scholars and researchers in China have done relevant research. Song Yan pointed out that the availability of drugs in public medical institutions in Shandong Province has been stable in the past 5 years, and the overall level of availability is not high, but it is still better than the average of middle and high income regions level [3]. The availability of essential medicines has a downward trend, and the availability of original medicines and low-cost medicines continues to increase. The policy on essential medicines was slightly loosened, and the dividends on the price policy of new medicines have appeared. Jiang Xiaotong pointed out that under the premise that the price and affordability of basic medicines are good, the availability of basic medicines in primary medical institutions in Liaoning Province is the lowest, especially for the treatment of chronic diseases [4]. This not only does not reflect the characteristics of fair access to essential medicines, but also violates the original intention of the “strong grassroots” of the essential medicine system, and is not conducive to the further promotion of graded diagnosis and treatment. It is recommended that relevant departments give priority to strengthening the supply and guarantee system of essential medicines in primary medical institutions. Chen Chen pointed out that the formulation of the basic medicine list in Hubei Province is not perfect, and the degree of compliance with the actual needs is not high, and the availability of medicines in Hubei Province is low [5].

The scarcity of resources determines the priority to meet basic needs and improve the efficiency of resource use at all times. It is necessary to ensure the availability of basic medicines to help meet the basic needs of the people’s most basic medicines, and promote the rational use of medicines to improve the effectiveness of health resources. Anhui Province is the earliest province in China to carry out medical policy reforms. We selected Hefei, the capital of Anhui Province, to conduct research. The purpose of this study is to investigate and analyze the availability and availability of essential medicines in public medical and health institutions and social retail pharmacies in Hefei. Affordability and provide a basis for subsequent formulation and improvement of relevant policies.

## Methods

### Survey objects and methods

#### Survey time

The survey period for this study was from June 2019 to October 2019.

### Survey selection of areas and institutions

The 2018 edition of the National Essential drugs List came into effect in September 2018. Relevant policies require that all primary medical and health institutions should supply national essential drugs, while secondary and tertiary medical and health institutions should supply a certain percentage of essential drugs.

According to the World Health Organization (WHO) and the International Health Action Organization (HAI) ‘s standard survey method for drug accessibility research, the scope of this survey must first be determined, that is, a national survey or a regional survey. For countries with large land areas or large populations, we can choose to conduct surveys based on regions. Considering the area and administrative divisions of Hefei City, this survey selected 4 main urban districts and 2 municipal counties in Hefei, namely Yaohai District, Baohe District, Shushan District, Luyang District, Feidong County, and Feixi County, a total of six. Selection of survey institutions: (1) Select a major public hospital (usually a public tertiary hospital) in each survey area. (2) Six medical institutions were randomly selected within a three-hour drive of the six public hospitals initially selected in the first step. According to this requirement, 4 medical districts and 2 affiliated counties should choose 30 medical institutions. (3) Randomly choose 30 retail pharmacies nearest to the selected public hospital.

### Investigating choice of disease type

According to the Fifth National Health Service Survey and Analysis Report 2013, the top five diseases with high prevalence in China are hypertension, colds, diabetes, gastroenteritis, and cerebrovascular disease [6].

#### Survey choice of drugs

WHO / HAI published the second edition of the “Survey Guidance Manual” in 2008, which clearly defined the drug survey catalogue. Considering the drug habits of different countries, the convenience of the survey, and the representativeness of the drugs, the survey was conducted with drugs divided into three categories: the international core drug list, the regional core drug list, and the supplementary list. In this study, while using the WHO / HAI Essential Drug Core List, combined with the basic situation of Hefei, a supplementary list was added based on expert consultation, and a total of 50 types of drugs were investigated.

### International drug Core list

The so-called International Drug Core List is the drug selected by WHO / HAI according to the disease spectrum and necessary for basic medical care worldwide. It is the safest, most effective and most economical drug. As the international core list is highly representative and convenient for comparison worldwide, this study included all 14 drugs in the international core list into the questionnaire. See Table 1.

**Table 1:** International core list of this questionnaire (international drug control group)

Serial number	General name	Dosage form
1	Amitriptyline	tablets / capsules
2	Amoxicillin	tablets / capsules
3	Atenolol	tablets / capsules
4	Captopril	tablets / capsules
5	Ceftriaxone	Injection
6	Ciprofloxacin	tablets / capsules
7	Compound Sulfamethoxazole	Suspension
8	Diazepam	tablets / capsules
9	Diclofenac	tablets / capsules
10	Glibenclamide	tablets / capsules
11	Omeprazole	tablets / capsules
12	Acetaminophen	tablets / capsules
13	Salbutamol	aerosol
14	Simvastatin	tablets / capsules

### WHO / HAI regional core drug list

Due to ethnic differences in various regions of the world, people in each region have different medication habits. In order to make the types of drugs surveyed more representative, WHO / HAI divides the world into six regions, including the Middle East, the Eastern Mediterranean, Southeast Asia, the African Region, the Caribbean Rim, and the Western Pacific Region. Each of these six regions has an independent regional core catalogue, each of which contains 16 surveyed drugs. China is divided into the Western Pacific region. In order to make the drugs in the questionnaire more representative of China ‘s medication habits, and to facilitate regional comparison with countries in the West Pacific region, the questionnaire selected 16 core drugs in the Western Pacific region. See Table 2.

**Table 2:** Core drugs list in the survey area

Serial number	General name	Dosage form
1	Metformin	tablets / capsules
2	Ibuprofen	tablets / capsules
3	Metronidazole	tablets / capsules
4	Dexamethasone	Injection
5	Fluconazole	tablets / capsules
6	Clarithromycin	tablets / capsules
7	Azithromycin	tablets / capsules
8	Aspirin	tablets / capsules
9	Glipizide	tablets / capsules
10	Carbamazepine	tablets / capsules
11	Nifedipine	tablets / capsules
12	Cefuroxime	tablets / capsules
13	Phenytoin sodium	tablets / capsules
14	Aminophylline	tablets / capsules
15	Fluoxetine hydrochloride	tablets / capsules
16	Erythromycin	tablets / capsules

### Supplementary catalog

In order to make the types of selected survey drugs more comprehensively represent China’s medication habits and disease spectrum characteristics, on the basis of consulting front-line experts in pharmacy, health management, and medical work, according to common diseases and medication habits in Hefei, another selected 20 drug was added to the questionnaire as supplement. All selected drugs are from the National Essential Drug List issued by China in 2018, see Table 3.

**Table 3:** General Catalog

Serial number	General name	Dosage form
1	Salbutamol sulfate	aerosol
2	Metformin hydrochloride	tablet
3	Bisoprolol fumarate	tablet
4	Captopril	tablet
5	Simvastatin	tablets / capsules
6	Amitriptyline hydrochloride	tablet
7	Ciprofloxacin	tablet
8	Compound Sulfamethoxazole	suspension
9	Amoxicillin	capsule
10	Dexamethasone	injection
11	Omeprazole	tablets / capsules
12	Diazepam	tablet
13	Oseltamivir	tablets / capsules
14	Acetaminophen	tablets / capsules
15	Diclofenac sodium	tablets / capsules
16	Atenolol	tablet
17	Glimepiride	tablets / capsules
18	Clarithromycin	tablets / capsules
19	Loratadine	tablets / capsules
20	Fluconazole	tablets / capsules
21	Hydrochlorothiazide	tablet
22	Azithromycin	tablets / capsules
23	Amlodipine	tablet
24	Digoxin	tablets / capsules
25	Glibenclamide	tablets / capsules
26	Metronidazole	tablet
27	Nifedipine (sustained release)	tablet
28	Diphenhydramine hydrochloride	tablets / capsules
29	Doxycycline hydrochloride	tablets / capsules
30	Ibuprofen	tablets / capsules
31	Irbesartan	tablets / capsules
32	Losartan potassium	tablets / capsules
33	Cefuroxime	tablets / capsules
34	Enalapril maleate	tablets / capsules
35	Fluoxetine hydrochloride	tablets / capsules
36	Gliclazide	tablets / capsules
37	Levofloxacin	tablets / capsules
38	Chlorpheniramine maleate	tablets / capsules
39	Atorvastatin calcium	tablets / capsules
40	Clomipramine hydrochloride	tablets / capsules
41	Glipizide	tablets / capsules
42	Ceftriaxone	injection
43	Propranolol hydrochloride	tablets / capsules
44	Erythromycin	tablets / capsules
45	Mupirocin	Ointment
46	Cefalexin	tablets / capsules
47	Carbamazepine	tablets / capsules
48	Clopidogrel hydrogen sulfate	tablets / capsules
49	Phenytoin sodium	tablets / capsules
50	Aminophylline	tablets / capsules

### Catalog adjustment instructions

The essential medicine survey catalogue has detailed regulations on drug dosage forms and drug specifications, but it does not meet the actual situation of medication in China [7]. If the prescribed drug specifications are fully adopted for the survey drug, there may be cases where the survey institution has a drug and the survey result is 0, which will greatly affect the accuracy of the survey results of the availability of essential drugs in China. This has happened in previous studies. Therefore, in this study, while conducting surveys in accordance with the requirements of the survey guidelines, only the dosage form of the target drug was restricted, and the drug specifications were actually filled in by the respondents in the survey.

The target drugs for the survey are OBs and generics under the general name of the same drug. The original research pharmaceutical company is the earliest manufacturer of this general name drug, also the developer of this drug, and has early patents for this drug. In the same surveyed institution, there may be cases where the same general name drug is produced by multiple manufacturers at the same time. Then the survey will preferentially record generics that are consistent with the WHO / HAI designated specifications. The drug is recorded according to the principle of the lowest price.

In addition, when investigating selected drugs, the OB with the same specifications and the lowest-priced generic drug should be used for comparison, and their availability and affordability in medical and health institutions and retail pharmacies should be examined. Originator Brands (OBs): Refers to drugs of the same specifications produced by the original manufacturer of the drug under survey. Lowest-priced generics (LPGs): Generics with the lowest unit price found in research institutions.

#### Evaluation index

##### Availability evaluation index

Availability of essential drugs refers to the proportion of the surveyed institutions that can provide a certain drug to the total number of survey institutions [8]. The formula for calculating availability is as follows: Availability = number of institutions that can provide medicines / total number of research institutions × 100%. Evaluation criteria: quite low, < 30%; low, 30 to 49%; relatively high, 50 to 80%; high, > 80%.

##### Affordability evaluation indicators

WHO / HAI ‘s definition of affordability of essential drugs: within a certain course of treatment, the total medicine cost for the treatment of a disease with standard doses of medicines is equivalent to the minimum daily salary standard for non-technical personnel in government departments (or equivalent to local Human resources and social security departments set minimum daily wage rates) multiples [9]. The survey showed that the minimum wage for employees in Hefei in 2018 was RMB 1520 / month, and the average daily salary was RMB51. According to the National Essential Drug Formulary (2018 Edition), the treatment cycle for acute infectious diseases is generally 7 days, and the treatment cycle for adult chronic diseases is generally 30 days.

Affordability evaluation criteria: If the total cost of drug treatment is lower than the above minimum daily wage standard, the drug is considered to be more affordable, and vice versa.

##### Median Price ratio of essential drugs

The median price ratio (MPR) refers to the ratio of the median price of a drug unit (tablet, capsule, doses, etc.) to the corresponding national reference price in a surveyed area [10]. It is not only an important indicator to measure the availability of essential drugs, but also an important indicator to evaluate the price level and international reference level of medicines in the survey area. The specific calculation formula of MPR is as follows: MPR = median unit price of the target drug within the survey range / international reference price × 100%.

It can be seen from the above formula that the calculation results of MPR values are closely related to international reference standards, and the international reference prices are displayed in US dollars with the data from the Health Management Science (MSH) International Price Guide. Therefore, according to the WHO Survey Manual, when the unit price of the target drug is recorded in the data, all are converted into US dollars according to the exchange rates of RMB and US dollars on the survey starting date and then compared. In order to ensure the reliability of the data, this study stipulates that the calculation of the median unit price of a drug can only be adopted if the sample size of the same general drug unit price is ≧ 5.

When using MPR values to compare drug purchase price levels, MPR = 1 is usually set as the threshold value. When this value is less than 1, it indicates that the price of the investigated drug is lower than the international average standard, and vice versa. For retail prices, the threshold of the MPR = 1 is too low to be reasonable. The retail price of the drug recommended by the World Health Organization(WHO) MPR < 2 is a reasonable retail price, while further research by Susanne Gelders et al. distinguishes the MPR of drug prices in retail pharmacies and medical institutions, and considers that reasonable drug prices in retail pharmacies was MPR < 2 and medical institutions MPR < 1.5. See Table 4. The following criteria are used in this study to determine the rationality of essential drugs’ price:

**Table 4:** Acceptable range of MPR

Analysis object	Acceptable range
Retail prices of essential drugs in medical institutions	MPR < 1
Retail pharmacy retail prices of essential drugs	MPR < 1.5

As the surveyed drugs did not regulate the dosage forms and specifications (mentioned above), considering the consistency and comparability of the survey data, in the data entry process of this study, the drug specifications for different dosage forms between the same general name were converted and unified into the specifications required for the first volume of the WHO survey. The conversion method adopts the rules for the approximate conversion of drug unit prices and specifications in China’s Drug Price Comparison Rules.

##### Quality control

In the initial stage of the survey, based on consulting the front-line experts of Hefei Health Service, the research team made several changes to the setting of the questionnaire to ensure the rationality and representativeness of the questionnaire. Prior to the survey, the research team provided investigators with relevant training on basic drug policies to ensure that there were no inaccuracies in the survey data due to the investigator’s personal problems during the in-site survey. Meanwhile, a small-scale pre-survey was conducted in the center of the survey area, and some settings of the questionnaire were adjusted based on the results of the pre-survey.

During the in-site survey, the research was led by the staff from the Hefei health administrative department, which received strong support from the regional health administrative department, and the accuracy of the survey data was guaranteed to the greatest extent possible.

#### Survey implementation and data statistics

Before implementation of the survey, the investigator formulates the availability and affordability survey forms based on the identified drugs, visits selected surveyed institutions, fills the data results into the form, and finally uses Excel software for data input and statistical analysis [11].

## Results

### Questionnaire recovery

A total of 60 valid questionnaires were collected, including 6 from tertiary class A hospitals, 24 from secondary hospitals, and 30 from retail pharmacies.

### Availability of essential drugs

See Table 5 for the comparison of the availability of essential drugs in tertiary class A hospitals and retail pharmacies. In general, the proportion of generics in public medical institutions is lower than that in retail pharmacies [12]. The median of generic drug allocation rate in public medical institutions was 32.3%, and the standard deviation was 0.2219, while the median of generic drug allocation rate in retail pharmacies was 56.8%, and the standard deviation is 0.1837. See Table 5.

**Table 5:** Generic Drug Allocation Ratio of Essential Drugs in Public Medical Institutions and Retail Pharmacies

	Median(M)	Standard deviation(S)	Upper quartile P25	Lower quartile P75
Public medical institution(*N* = 30)	32.3%	0.2219	18.7%	58.2%
Retail pharmacy(N = 30)	56.8%	0.1837	41.8%	62.1%

### OBs supply in institutions

Among the investigated samples, the OBs proportion in medical institution or retail pharmacy was quite low. The OBs proportion in all drug sales institutions did not exceed 25%.

### Drug availability

#### Drug availability in retail pharmacies

Of the 14 drugs in the WHO International Core List, 7 drugs have a allocation rate of more than 50%. The generics are: amoxicillin, captopril, diclofenac, omeprazole, acetaminophen, and salbutamol and simvastatin. Among them, the omeprazole allocation rate reached 86.7%, which means that there are 26 out of 30 pharmacies supply omeprazole generics. There are 7 drugs with a allocation rate of less than 50% and low availability. Among them, the allocation rate of amitriptyline and atenol is 0, and no pharmacy sells this drug. See Table 6.

**Table 6:** International Core List Drug Allocation Rate

Serial number	General name	Allocation rate
1	Amitriptyline	0
2	Amoxicillin	80%
3	Atenolol	0
4	Captopril	76.7%
5	Ceftriaxone	40%
6	Ciprofloxacin	33.3%
7	Compound Sulfamethoxazole	26.7%
8	Diazepam	46.7%
9	Diclofenac	70%
10	Glibenclamide	33.3%
11	Omeprazole	86.7%
12	Acetaminophen	83.3%
13	Salbutamol	70%
14	Simvastatin	53.3%

Of the 20 drugs in the survey supplementary list, 6 drugs were poorly available. Among them, glipizide, carbamazepine, aminophylline, and fluoxetine hydrochloride were quite low. Aminophylline supply with a rate of 0 and is not sold in all surveyed pharmacies. Among the more available drugs, total five drugs have a allocation rate of more than 70%: metformin, aspirin, nifedipine, metronidazole, and azithromycin.

#### Drug availability in medical institutions

In the survey of medical institutions, it was found that there were 5 drugs with high availability of generics, such as omeprazole, accounting for 10% of the total, of which omeprazole has the highest ratio for 90%. Metformin, aspirin, and acetaminophen with allocation rates exceeding 80%. There are 12 varieties with high availability, accounting for 24% of the total. Among them, amoxicillin, oseltamivir, amlodipine besylate, irbesartan, chlorpheniramine maleate, and atorvastatin calcium exceeded 60%. Nine drugs have low availability, and fluoxetine is not supplied in all surveyed medical institutions with 0 availability.

#### Availability comparison of generics in public medical institutions and retail pharmacies

Including oseltamivir, aspirin, amoxicillin, amlodipine besylate, omeprazole, and clopidogrel bisulfate, the six drugs have high allocation rates in medical institutions and retail pharmacies.

#### Availability of OBs

The availability of OBs is quite low in medical institutions nor retail pharmacies. The survey collected data on 14 OBs, including amoxicillin, captopril, ceftriaxone, diclofenac, omeprazole, salbutamol, simvastatin, metformin, ibuprofen, azithromycin, aspirin, nifedipine, carbamazepine, and fluconazole. Among them, the OB of ceftriaxone and fluconazole is only available in public medical institutions, while the OB of captopril can only be found in retail pharmacies. Of the 14 OBs, the OB availability of metformin and aspirin was more than 50%, and the availability of the other 12 drugs was quite low with availability less than 50%. Omeprazole and azithromycin, the two OBs, have significantly higher allocation rates in retail pharmacies than in public medical institutions.

### The price level of essential drugs in Hefei

#### MPR analysis of OBs in retail pharmacies

Total 14 OBs purchase and retail prices were collected in all investigating institutions, of which only diclofenac, azithromycin, metformin, omeprazole, carbamazepine, aspirin, nifedipine, and salbutamol with sampled amount greater than 5. The median MPR value of the eight OBs is 7.85, which means that the OB price is 7.85 times the international reference price.

#### MPR value analysis of generics in retail pharmacies

The price of 21 generics that meet the calculation standards is generally lower than the international level, and its median MPR value is 0.9, indicating that it is only 90% of the international level. The MPR values in the International Core Directory and Regional Core Directory are respectively 0.9 and 0.8. Among these 41 drugs, the maximum value of MPR is metronidazole-2.1, and the minimum value is azithromycin-0.2, which is equivalent to 20% of the international standard drug price, see Table 7. Total 17 generics have MPR values below 1.5, which means that 81.1% of generics are acceptable within a reasonable range. Another 4 generics have higher MPR values than the international level.

**Table 7:** General MPR of generics in retail pharmacies

	quantity	median(M)	P25	P75	maximum	minimum
International Core Directory	10	0.9	0.6	1.3	1.7	0.2
Regional Core Directory	11	0.8	0.5	1.1	2.1	0.2
Total	21	0.9	0.6	1.2	2.1	0.2

#### MPR analysis of essential drugs in public medical institutions

Among the domestic generics of public medical institutions, only enanpril, metformin and amoxicillin had MPR values significantly higher than 1, and higher than international levels. The prices of most other domestic generics are controlled between 0.3–0.8, and retail prices are significantly lower than international standards. The MPR value of the OB in public medical institutions is significantly higher, the MPR value of omeprazole reached 5.2, the MPR value of amoxicillin reached 10.6, and the OB price of captopril reached 18.3.

#### MPR value comparative analysis of essential drug in public medical institutions and retail pharmacies

Comparing 21 generics and 4 OBs in the two types of medical institutions, the comparison found that the drug prices of medical institutions are generally lower than retail pharmacies with significant differences. Among the 4 OBs, omeprazole and aspirin have similar prices between retail pharmacies and medical institutions. Amoxicillin and captopril have higher prices in medical institutions than retail pharmacies, which are 2.4 times and 1.3 times respectively.

Among the 21 generics surveyed, only the medical institutions’ price of amoxicillin and enalapril had higher retail prices than in pharmacies. The pharmacy prices of omeprazole and aspirin were the same as those of medical institutions. The prices of the remaining 17 domestic generics in public medical institutions are lower than retail pharmacies in various degree.

### Affordability of essential drugs in Hefei

The survey found that 13 drugs for 7 categories including respiratory diseases, gastrointestinal diseases, antimicrobials, hypertension, hyperlipidemia, diabetes, and asthma were available in public medical institutions and retail pharmacies.

Comparing the price of medicines required for a single course of treatment (daily cost ×treatment duration) with the minimum wage of a worker in Hefei City (RMB51/ day), it was found that the total cost of a single course of treatment for 5 drugs was lower than the average daily salary of Hefei workers. The overall affordability of essential drugs in Hefei is better.

## Discussion

To authors’ knowledge, no scholar has done research on the availability and affordability of essential drugs in Hefei before, and some scholars have discussed the availability and affordability of essential drugs in cities such as Hangzhou, Baoji, and Shanghai.This article aims to ascertain the specific situation of the availability and affordability of essential drugs in Hefei, and to make relevant suggestions to improve the current situation. This article mainly compares key factors such as drug prices, allocation rates, and median price ratios, focusing on the public medical institutions and retail pharmacies [13].

The survey results in this article focus on the availability and affordability of medicines. 30 public medical institutions and 30 retail pharmacies were selected as survey samples [14]. The results show that the essential drug allocation rate for public medical institutions and retail pharmacies is low in Hefei with the lower drug availability. The national essential drug system has been implemented in Hefei City and has achieved certain results since its implementation. However, there is still a gap from the expected target, which needs to be continuously explored and improved in practice.

Before implementing the centralized drug procurement policy, the government has been looking for ways to increase the efficiency of drug distribution and reduce drug delivery time. At present, the expected results have not been achieved [15]. There are two possible explanations. One possibility is that the purchase of retail pharmacies has reached saturation. Even if there is no relevant policy, the purchase volume of retail pharmacies can only reach one degree. Another explanation may be that the centralized procurement policy has a negative impact on retail pharmacies, and the policy hinders the purchase of medicines [16].

### Allocation analysis of essential drugs in Hefei

Essential drugs are the drugs most closely related to the patients’ lives, and in the ideal state, drug sales institutions should supply to the maximum extent. However, in Hefei, whether in public medical institutions or retail pharmacies, the allocation of essential drug is not satisfactory [17]. This study found that 50% of essential drugs at public medical institutions and retail pharmacies are quite low, and some medicines are hard to find in the market. The low allocation rate of essential drugs leads to the fact that patients do not enjoyed the benefits of low prices of essential drugs because they choose high-priced equivalent drugs in actual purchase.

The allocation ratio of OBs in retail pharmacies and public medical institutions is not high, and the both allocation ratio is basically the same. In terms of domestic generics, its sales in retail drug stores in Hefei are better than that of public medical institutions. The median allocation rate of essential drug in retail pharmacies is 60.0%, which is significantly higher than 30.0% in public medical institutions.

In China, the essential drug system is in an exploratory stage, and the system itself has not yet reached a perfect stage. Some medicines are expensive and unavailable, not included in the procurement catalogs of public medical institutions and retail pharmacies, resulting in low availability of medicines. In fact, there are alternative medicines, and the indications for the treatment of similar varieties are the same, and some public medical institutions and retail pharmacies purchased the alternative medicines instead of drugs in WHO list, resulting in lower availability of this drugs. The availability of medicines will be improved if factoring in alternative medicines is taken into account. Due to the influence of price factors, after consulting and communicating with patients, doctors and pharmacists may choose low-cost alternative medicines for them, resulting in lower availability of medicines.

Beginning in 2018, in order to stabilize China’s pharmaceutical market, the Chinese government began to implement the “4 + 7 Volume Procurement Policy.” It refers to the state-approved centralized drug procurement at selected cities, covering Beijing, Tianjin, Shanghai, Chongqing, Shenyang, Dalian, Xiamen, Guangzhou, Shenzhen, Chengdu, Xi’an, etc. 11 cities. Such policies have led to a substantial drop in drug prices, for example, the price of therapeutic drug entecavir dispersible tablets fell by 92% with an average decline of more than 50%. In the bidding procurement in 2019, the “4 + 7 Volume Procurement Policy” model is also tracked in areas out of the selected cities. It is generally believed that the prices of essential drugs will continue to fall. After the “4 + 7 Volume Procurement Policy”, there has been a trend to reduce prices, reducing the burden on patients, but the drugs shortage is also increasing. For example, the Chinese government has established a drug shortage monitoring mechanism, and provinces have also published their own drug shortage catalogs.

The purpose of the Chinese government’s “4 + 7 Volume Procurement Policy” is to reduce the drug price and improve the patients’ purchase ability. Successful bidders who pass the conformity assessment of generics will greatly reduce drug prices. The government will give the successful bidders a certain market share, and the coverage of high-quality and low-cost generics will become wider. The pressure on the affordability of drugs will be reduced, and patients will have better drug options. Some pharmaceutical industry associations have applied to suspend the implementation of the policy, fearing that excessive price declines will affect the profitability of pharmaceutical companies, reduce research and development investment, and affect drug innovation. In the long run, patients will lose new treatments, which is not good for them, but the medical insurance department is determined to implement the policy in order to save medical insurance funds. For business risks control, pharmaceutical companies have adopted methods to reduce manufacturing investment and strictly control the scale of drugs production. Due to China’s huge region and the uneven development between provinces, a unified market circulation system has not yet been formed in China. When market fluctuations occur, there may be a decline in the supply of medicines in some regions or within a certain period of time, which reduces the availability of drugs [18].

Some medicines, such as metformin, amoxicillin, and nifedipine, have large stocks in public medical and retail pharmacies. Due to cheaper generics and market factors, public medical institutions and retail pharmacies will give priority to purchasing generics, such as omeprazole. Its availability is high, and its ratio of the OB and the generics is over 80%. However, most public medical institutions and retail pharmacies have more stocks of generics than stocks of OBs, which may be related to economic factors and policy factors. Pharmaceutical manufacturers may have economic interests with public medical institutions and retail pharmacies, so they will give priority to purchasing generics [19].

### Affordability analysis of essential drugs’ retail prices

The affordability of medicine prices is an important guarantee for the availability of medicines. The affordability of most essential drugs in Hefei is good. Hefei is a relatively underdeveloped region in East China. The per capita income in urban and rural areas is much lower than in economically developed provinces, such as Zhejiang, Jiangsu, etc., and to a certain extent, higher drug expenditures will cause patients to be “poor due to illness” or “back to poverty due to illness” [20].

By 2018, the Hefei Municipal Government has formulated relevant policies to control drug prices and drug procurement scope. From the survey results, the affordability of public medical institutions is better than that of retail pharmacies. However, the prices of some drugs are still higher than the international standards set by WHO / HAI, and they still need to be improved. Generally, the release and implementation of policies will benefit the availability and affordability of drugs, but the actual situation is not optimistic [21]. It is recommended that government departments strengthen drug price supervision to make the procurement system more scientific, operate more efficiently, and make the circulation open and transparent.

In the future, due to the influence of national policies, the affordability of drugs in Hefei City will continue to remain at a better level, and low-cost OBs and high-quality generics will enter public medical institutions and retail pharmacies. For patients, it can solve related drug procurement issues [22].

In the drug affordability survey standards formulated by WHO / HAI, it is defined as the total cost of medicines used to treat a disease with a standard dose of medicines within a certain course of treatment, which is equivalent to the minimum daily salary standard for non-technical personnel in government departments (Or equivalent to the minimum daily wage standard set by the local human resources and social security department). There is a certain deviation from the statistics of Chinese residents’ income. Some residents are individualized or farmed, and cannot be evaluated according to the standards set by WHO / HAI.

This research also has some disadvantages. The first is the time lag of research work. Recently, the Chinese government’s medical insurance drug procurement policy is undergoing a drastic changes, and it can be said that there will be substantial changes every few months. For example, on November 28, 2019, the new national medical insurance catalog has just launched, and the prices of most essential drugs and new medicines have been significantly reduced in order to enter the medical insurance catalog. However, some essential drugs are no longer included in the reimbursement list, and its prices may not be restricted, nor its production may halt due to sales decline. These changes will directly affect the availability and affordability of essential drugs. Second, there may be contingencies in actual investigations. The representativeness of selected hospitals and pharmacies may be biased, and accidental factors are not excluded from the statistical research results, which affect the final research results. Third, the core catalogue developed by WHO / HAI is based on global considerations without considering China’s national conditions. The drastic changes and uncertainties in China’s ongoing national healthcare reform and supporting pharmaceutical procurement reform have also affected the results of this study.

## Conclusions

The survey found that the availability of drugs in Hefei City is poor, and many OBs and generics are not supplied in public medical institutions and retail pharmacies. The affordability of most medicines are better, and the total cost of drug treatment is lower than the minimum daily wage standard in Hefei.

It can be said that the drug price factors are closely related to the national medical insurance policy directly affect the availability and affordability of drugs. In addition, China’s unique regional drug distribution management pattern, for Hefei or such cities or regions, requires more carefully consider the payment mechanism for medical insurance funds, drug prices, and the balance between affordability and availability. Strengthen the construction of grassroots medical and health institutions, scientifically select essential drug catalog varieties, rationally adjust drug prices, ensure the supply of low-cost drugs, popularize basic drug knowledge, and deeply implement the national essential drug system.

## Data Availability

All the medicines included in the study are listed in Table. Anhui Licensed Pharmacists Association.

## Abbreviations

WHO: World health organization
HAI: Health Action International
LPGs: Lowest-price generics
OB/Obs: Originator brands
MPR: The median price ratio
MSH: Health Management Science
